# Determination of carbamazepine in urine and water samples using amino-functionalized metal–organic framework as sorbent

**DOI:** 10.1186/s13065-018-0446-x

**Published:** 2018-06-30

**Authors:** Mohammad Reza Rezaei Kahkha, Ali Reza Oveisi, Massoud Kaykhaii, Batool Rezaei Kahkha

**Affiliations:** 1 Department of Environmental Health Engineering, Faculty of Health, Zabol University of Medical Sciences, Zabol, Iran; 2 Zabol Medicinal Plants Reseach Center, Zabol University of Medical Sciences, Zabol, Iran; 3 Department of Chemistry, University of Zabol, Zabol, Iran; 4 Department of Chemistry, Faculty of Sciences, University of Sistan and Baluchestan, Zahedan, Iran; 5 Smartphone Analytical Sensors Research Centre, University of Sistan and Baluchestan, Zahedan, Iran

**Keywords:** Carbamazepine, Pipette-tip solid phase extraction, Zirconium-based metal–organic framework, Urine analysis

## Abstract

A stable and porous amino-functionalized zirconium-based metal organic framework (Zr-MOF-NH_2_) containing missing linker defects was prepared and fully characterized by FTIR, scanning electron microscopy, powder X-ray diffraction, and BET surface area measurement. The Zr-MOF-NH_2_ was then applied as an adsorbent in pipette-tip solid phase extraction (PT-SPE) of carbamazepine. Important parameters affecting extraction efficiency such as pH, sample volume, type and volume of eluent, amount of adsorbent, and number of aspirating/dispensing cycles for sample solution and eluent solvent were investigated and optimized. The best extraction efficiency was obtained when pH of 100 µL of sample solution was adjusted to 7.5 and 5 mg of the sorbent was used. Eluent solvent was 10 µL methanol. Linear dynamic range was found to be between 0.1 and 50 µg L^−1^ and limit of detection for 10 measurement of blank solution was 0.05 µg L^−1^. This extraction method was coupled to HPLC and was successfully employed for the determination of carbamazepine in urine and water samples. The strategy combined the advantages of fast and easy operation of PT-SPE with robustness and large adsorption capacity of Zr-MOF-NH_2_.

## Introduction

Carbamazepine (CBZ, 5H-dibenzo [b,f] azepine-5-carboxamide) often used as anticonvulsant drug for treatment of epilepsy [1, 2]. Whenever a patient consumes CBZ, about 2–3% of this drug will excrete unchanged through his urine and enters into environmental aquatic ecosystems [3]. Studies confirmed that CBZ can be present in wastewater (up to 6.3 µg L^−1^) [4–7], surface water (up to 1.1 µg L^−1^) [8, 9], and drinking water (around 30 ng L^−1^) [10, 11]. Biodegradation of CBZ is very difficult in environmental media owing to its low solubility and stability in water. Therefore, several methods including advanced oxidation processes (AOPs), adsorption on various sorbent media have been employed for the removal and extraction of it [1, 2, 12–14].

In recent years, some sample preparation techniques such as liquid–liquid extraction (LLE) [15], dispersive liquid–liquid microextraction (DLLME) [16] and solid-phase extraction (SPE) [17] have been used for isolation and extraction of CBZ in complicated matrices. SPE is a prevalent procedure for pre-treatment of various pharmaceutical analytes due to its reproducibility, high recovery and simple operation. Miniaturized SPE has been developed to overcome on the problems raised by conventional SPE processes such as matrix effect, low detection limit, losses of analytes, and environmental problems due to consumption of large amounts of organic solvents.

Pipette-tip solid-phase extraction (PT-SPE) is a convenience, and microscale of SPE method which reduces amount of sorbent and reagents and saves the analysis time [18–20]. This technique required several repeated aspirating/dispensing cycles to complete the extraction procedure.

**Fig. 1:**
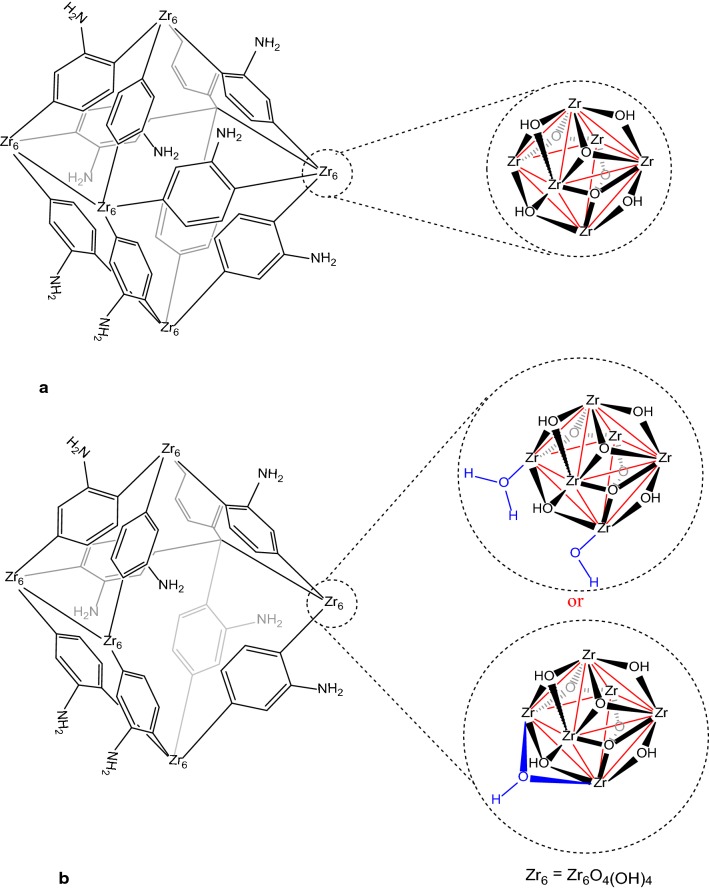
The idealized (**a**) and defective (**b**) structure of UiO-66-NH_2_

Metal–organic frameworks (MOFs), a new type of 3D crystalline porous materials assembled by metal ions (or clusters) and multi-topic organic ligands, have received significant attention in a wide array of potential applications such as photocatalysis [21, 22], gas storage [23, 24], separation [25, 26], drug delivery [27, 28], deactivation of chemical warfare agents [29, 30], conductivity [31, 32], removal of toxic materials [33, 34], and sensing [35, 36], due to their large porosity, very high surface area, tunable pore dimensions and topologies as well as their physicochemical properties [37]. Their well-ordered porous structures can create a unique microenvironment to enhance adsorption and penetration of guest species inside the frameworks. Zirconium-based metal–organic frameworks (Zr-MOFs) are one of the most promising MOF materials for practical applications, owing to their thermal, mechanical, and chemical stabilities besides their high surface area and low density. Zr-MOF-NH_2_ is an amino-functionalized Zr-MOF with the idealized chemical formula Zr_6_O_4_(OH)_4_(L)_6_ (L = 2-aminoterephthalate) and uniform three-dimensional pores structure composed of 2-aminoterepthalate linkers and hexanuclear [Zr_6_(*μ*_3_–O)_4_(*μ*_3_–OH)_4_]^12+^ nodes, each connected to 12 carboxylates of the linkers to yield super octahedral and super tetrahedral cages/cavities (Fig. 1a) [38]. Recently, Hupp and Farha have reported a simple and producible procedure for the preparation of the Zr-MOF-NH_2_, which contains missing-linker defects [39]. The defects can result in the following advantages; (a) more hydroxyl groups and more open zirconium metal sites which could increase analyte binding affinity and selectivity, and (b) large pores and apertures which might lead to enhance substrate transport rates and in some cases selectivity (Fig. 1b). These advantages combined with amino functionality on organic linker (as coordinating and hydrogen-bonding sites via amino group in addition to possibility of the non-covalent interactions between the organic aromatic linker and guest species) could further improve separation performance and selectivity of the MOF [40–44].

Intrigued by the above-mentioned findings, we encouraged to prepare and use the bio inspired sponge, amino-functionalized Zr-MOF, for micro-scale solid phase extraction and determination of the carbamazepine. Several parameters affecting extraction efficiency including pH, type and volume of eluent, volume of sample solution, and amount of sorbent, number of draw/eject of sample solution and eluent solvent type were tested and optimized. Finally, the method was used for the determination of carbamazepine in urine and water samples.

## Experimental

### Chemicals and materials

All reagents (analytical grade) were purchased from Sharloa (Spain) and used as received, except HPLC solvents which were of chromatographic grade. All aqueous solutions were prepared using ultra-pure Milli-Q^®^ purification system. 20 µL pipette-tips (Dragon Lab, China) were used as micro columns. Carbamazepine was obtained from Sigma-Aldrich (St. Louis, MO, USA).

### Synthesis of Zr-MOF-NH_2_ sorbent

Zr-MOF-NH_2_ was synthesized according to the Hupp/Farha method [42] with minor modifications. In a 25 mL vial, dimethyl formamide (5 mL) and concentrated HCl (2.85 mL, 850 mmol) were added to 0.125 g, (0.54 mmol) of ZrCl_4_ before being sonicated for 10 min. A mixture of 2-aminoterephthalic acid (0.134 g, 0.75 mmol) and dimethyl formamide (10 mL) were then added to the clear solution and the mixture was sonicated for 20 more minutes. Afterwards, the capped vial was placed in a pre-heated oven at 80 °C for 15 h. After cooling to room temperature, the solid Zr-MOF-NH_2_ was filtered and washed with dimethyl formamide, and then with ethanol several times. In order to evaporate any solvents, this product was left for several hours under the hood and then was dried under reduced pressure (80 °C, 3 h). The solid Zr-MOF-NH_2_ was then activated at 120 °C for 12 h under high vacuum prior to measuring N_2_ isotherms.

### Characterization of Zr-MOF-NH_2_

Fourier-transform infrared spectroscopy (FT-IR) spectra were recorded using a Perkin-Elmer FTIR (USA). Powder X-ray diffraction (PXRD) patterns were recorded on a Philips X’pert diffractometer (Germany) with monochromated Cu Kα radiation (λ = 1.5418 Å) within the range of 1.5° < 2*θ* < 38°. Samples for scanning electron microscopy (SEM) were sputtered with a layer of Os (5-nm thickness) prior to taking images on a Hitachi S-4800 SEM (Japan) with a 15.0 kV accelerating voltage. BET surface area measurements were made at 77 K with liquid nitrogen on a Micrometrics TriStar 3020 (N_2_) surface area analyzer (Britain). Zr-MOF-NH_2_was degassed for 12 h at 120 °C before the measurement under a stream of nitrogen.

### Chromatographic analysis

Determination of CBZ was performed on an HPLC manufactured by Cecil company (England), equipped with a C_18_ ACE column (250 × 4.6 mm, 5 μm particle sizes) and a UV detector at wavelength of 210 nm. A mixture of water: acetonitrile (75:25) were used as mobile phase (isocratic elusion). Column was thermostated at room temperature. Injection volume and flow rate were 10 µL and 1 mL min^−1^, respectively.

### CBZ Extraction procedure

5 mg of Zr-MOF-NH_2_ was transferred to a 20 µL pipette-tip as micro column and attached to 100 µL variables sampler (Isolable, Germany). 100 µL sample solution was then introduced to column and passed over the sorbent and dispensed back to a 1 mL test-tube. The same sample solution was loaded into the micro column for 5 cycles. Adsorbed CBZ was then eluted by 10 µL of methanol in a 1 mL test-tube for 7 cycles, from which, 20 µL was injected to HPLC. Urine sample was collected from a healthy female and stored at − 80 °C and used throughout all experiments. This participant was not using supplements containing CBZ. Before start of the experiments, sample was brought to the room temperature, of which 250 µL was transferred to a canonical centrifuge tube. After addition of 1 mL of 1 M ammonium persulphate, it was heated in a water bath for 60 min at 95 °C. Then, this solution was brought to room temperature and was extracted by means of the suggested procedure. Tap water was obtained from laboratory and sample was filtered through a 0.45 µm Whatman filter paper and spiked with carbamazepine.

## Results and discussion

### Characterization of adsorbent

**Fig. 2:**
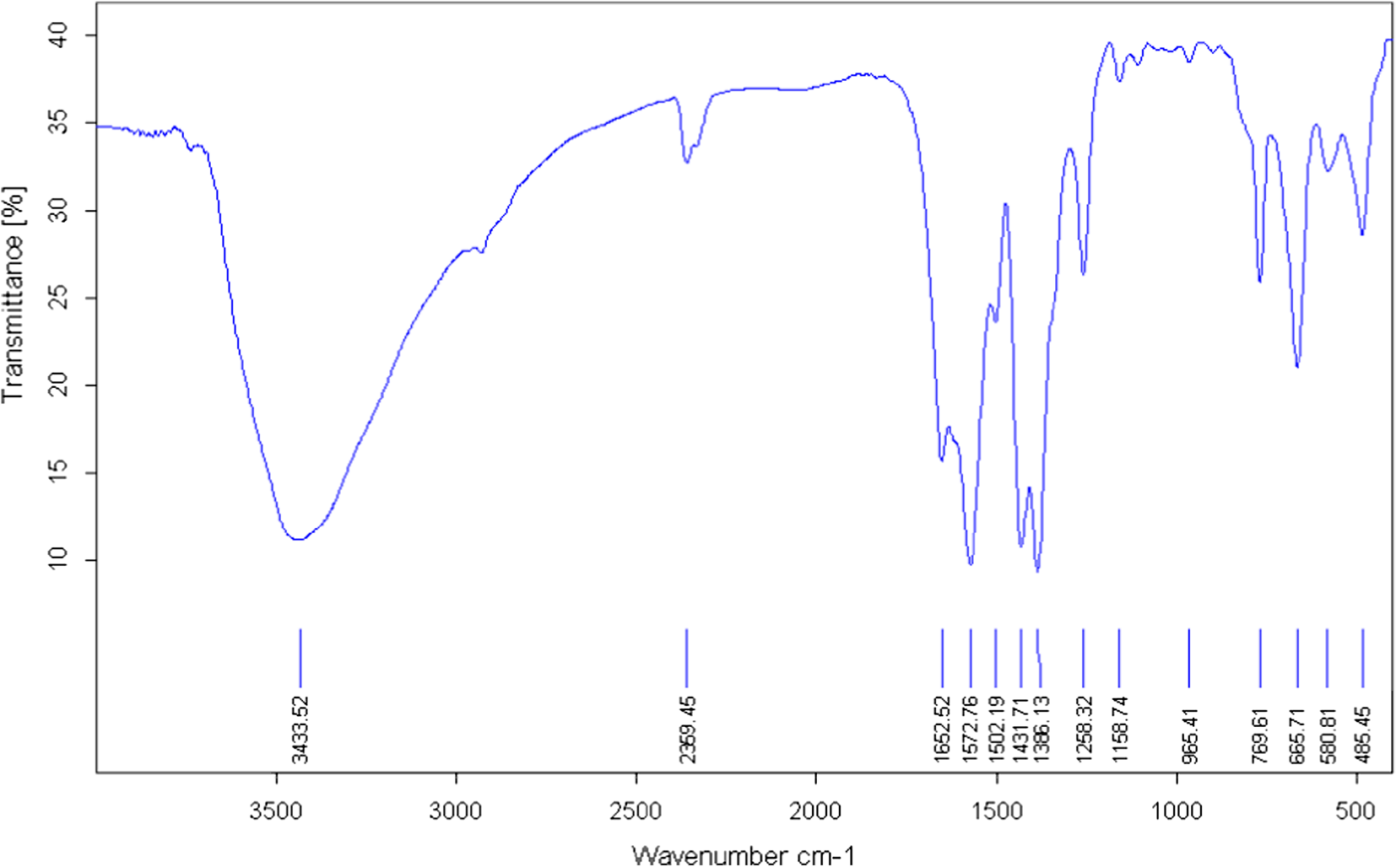
FTIR spectrum of synthesized Zr-MOF-NH2

**Fig. 3:**
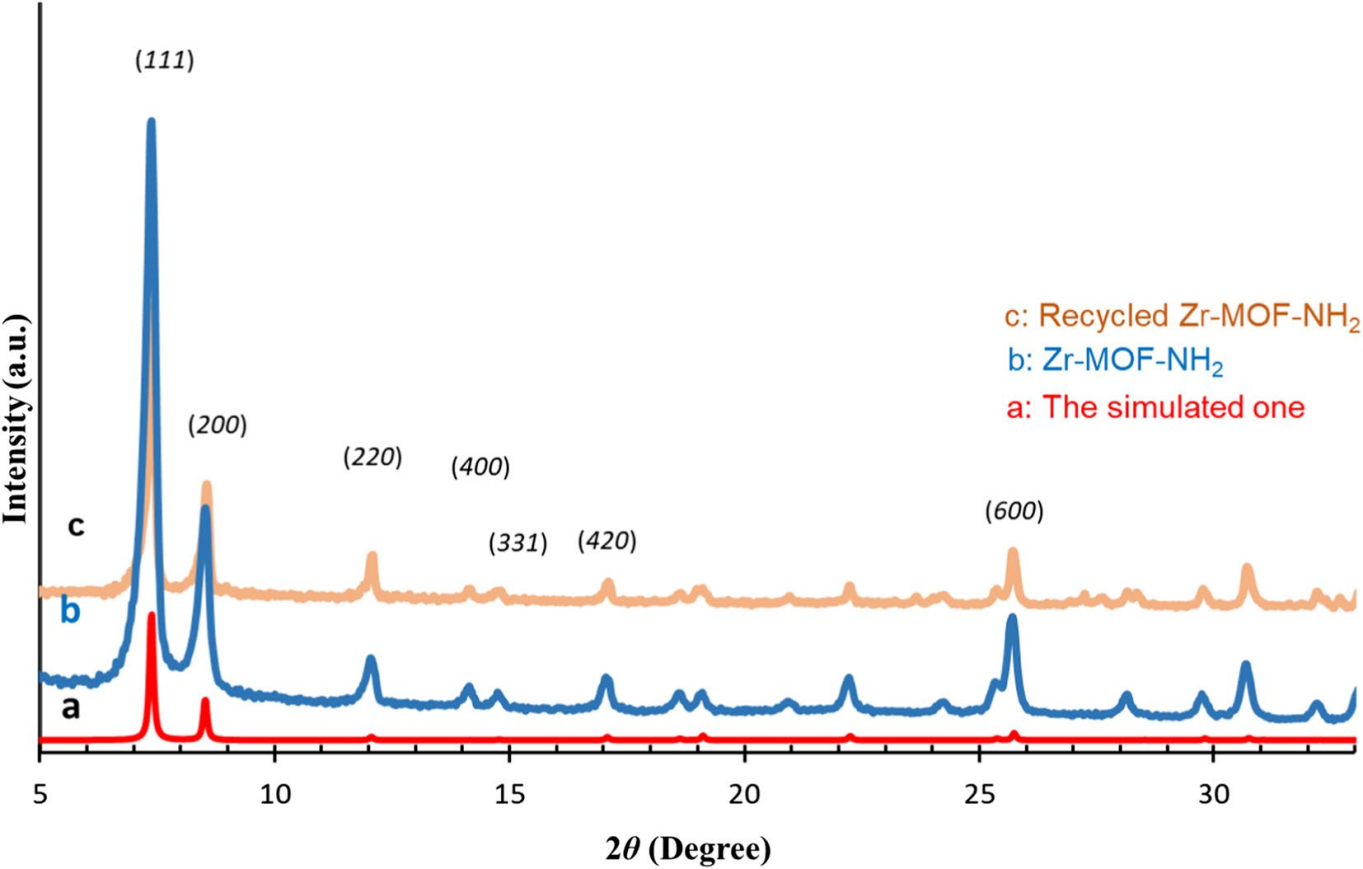
PXRD patterns of **a** the simulated Zr-MOF-NH_2_; **b** as-synthesized; and **c** the recycled Zr-MOF-NH_2_

Zr-MOF-NH_2_ was synthesized using 2-amino-terephthalic acid as the linker, zirconium (IV) chloride as the metal source and HCl as the modulator via a common solvothermal method (see the experimental section and Fig. 1). FT-IR spectrum of the Zr-MOF-NH_2_ shows a broad absorption peak (at about 3433 cm^−1^) related to the N–H (the asymmetric and symmetric) and O–H stretching modes (Fig. 2). The peak at 1654 cm^−1^ is assigned to DMF, while the intense doublet at 1572 and 1386 cm^−1^ are assigned to the asymmetrical and symmetrical stretching modes of the carboxylate groups (two strongly coupled C–O bonds with bond strengths intermediate between C=O and C–O). The strong aromatic C–N stretching band is observed at 1258 cm^−1^. The observed peaks between 1400 and 1500 cm^−1^ are ascribed to the C=C in aromatic compound of the organic linker. The peak at 769 cm^−1^ is assigned to C–C vibrational mode in the aromatic ring (Fig. 2). The powder X-ray diffraction (PXRD) pattern of the as-prepared Zr-MOF-NH_2_ agreed well with its structure reported in literature and the simulated PXRD pattern of UiO-66 [40–43]. The main peaks at 2θ = 7.3° and 8.5° are corresponded to the (*111*) and the (*200*) crystal planes, respectively (Fig. 3). The PXRD pattern of the Zr-MOF-NH_2_ is similar to the one described in literature, confirming the crystalline structure of the MOF. All 2θ peaks are in good agreement with that of PXRD patterns of the Zr-MOF parent material and the simulated one (CCDC No. 889529). The peaks at about 2θ = 7.3°, 8.5°, 12°, 17°, 18.6°, 19.1°, and 22.2° with d spacing of 11.9, 10.3, 7.3, 5.1, 4.7, 4.6, and 4.0 Å can be related to the (1 1 1), (2 0 0), (2 2 0), (4 0 0), (3 3 1), (4 2 0), and (6 0 0) reflections. The intensive peaks at 2θ = 7.3° and 8.5° are corresponded to the planes of tetragonal zirconia.

**Fig. 4:**
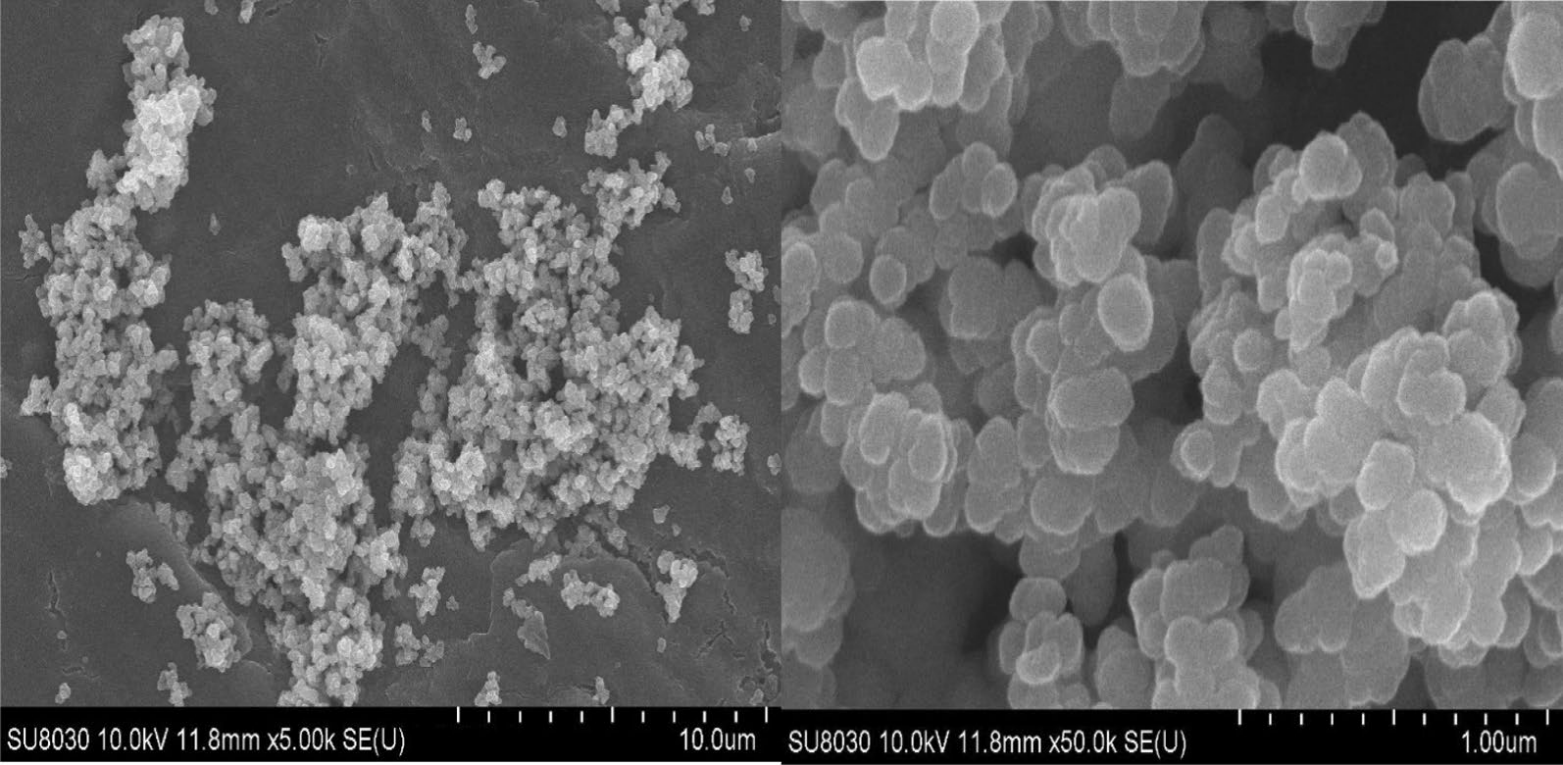
SEM images of the Zr-MOF

The morphology of the MOF was examined by scanning electron microscopy (SEM) (Fig. 4). Unlike the octahedral crystal shape of Zr-MOF-NH_2_ obtained by other methods [44], the SEM images of the nominal MOF showed aggregates of quasi-spherical particles between 100 and 200 nm.

**Fig. 5:**
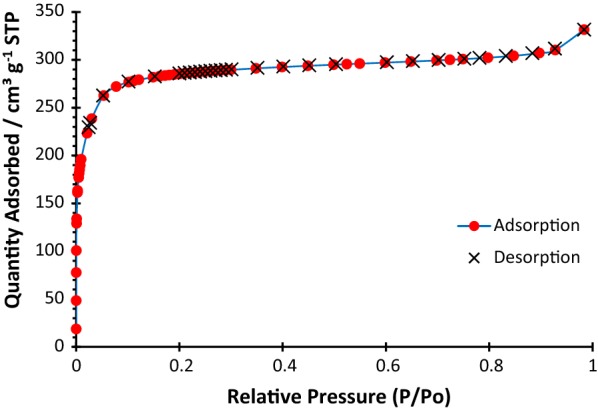
BET surface area measurement of Zr-MOF-NH_2_ at 77 K

The permanent porosity of Zr-MOF-NH_2_ was measured via nitrogen adsorption and desorption (Brunauer–Emmett–Teller, BET), indicating the highly accessible surface area of 1105 m^2^ g^−1^, and Langmuir surface area of 1319 m^2^ g^−1^, with a pore volume of 0.510667 cm^3^ g^−1^. Desorption average pore diameter was found to be 1.848 nm, and the average pore hydraulic radius was measured 0.3.787 nm (Fig. 5). The Zr-MOF-NH_2_ exhibited the type I isotherm which is characteristic of microporous materials.

### Optimization of PT-SPE procedure

To achieve the best extraction efficiency, we tried to optimize the conditions influencing the extraction processes as described below. All optimization experiments were performed with 10 µg L^−1^ of CBZ solution.

#### Effect of pH

**Fig. 6:**
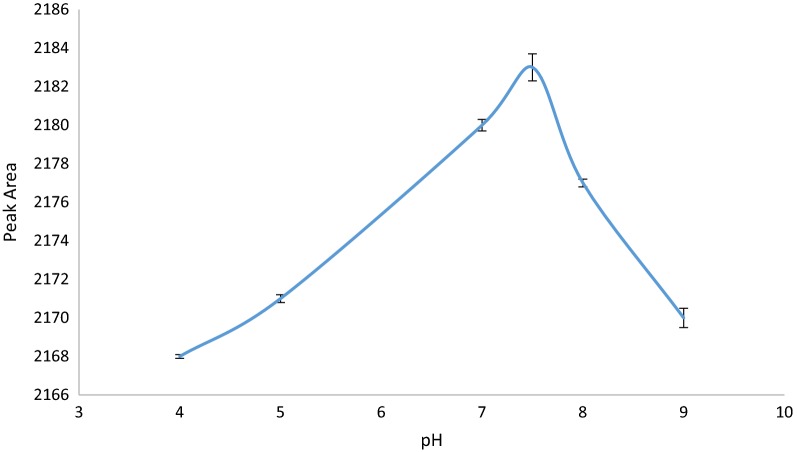
Effect of pH on extraction efficiency of CBZ (Experimental conditions: amount of adsorbent: 3 mg; sample volume: 150 µL; volume of eluent: 30 µL and number of draw/eject cycle for sample solution and eluent: 10 cycles)

pH is one of the most important factors in solid phase extraction. This factor illustrates how adsorption can be occurred and which form of the analyte (ionic or molecular) was adsorbed by the sorbent. For evaluation of the effect of pH on extraction efficiency, pH of samples was investigated between 4 and 9 and results are depicted in Fig. 6. As can be seen, the best pH value is 7.5 (around neutral pH) which indicates that CBZ adsorbs on Zr-MOF-NH_2_ by hydrogen bonding between the amino functionality and surface Zr–OH groups of MOF and carbamazepine. Moreover, Lewis acid–base interaction between CBZ and Zr-MOF-NH_2_ (including the zirconium ions as an open active sites and the free-carboxylate) may enhance adsorption. The increased affinity for CBZ observed in Zr-MOF-NH_2_ is a result of an increase in missing linker defects in the functionalized framework because of more terminal and sorbate-displaceable node hydroxo and free-carboxylate ligands. It should be noted that neutral pHs, terminal aqua ligands are mainly converted to hydroxo ligands; therefore, each missing linker generates a pair of defects (one on each node), with each defect site containing of a pair of hydroxo ligands bound to a single zirconium ion and a free-carboxylate group. The Zr-MOF with large numbers of defects can results in increasing capacity of CBZ adsorption.

#### Amount of adsorbent

**Fig. 7:**
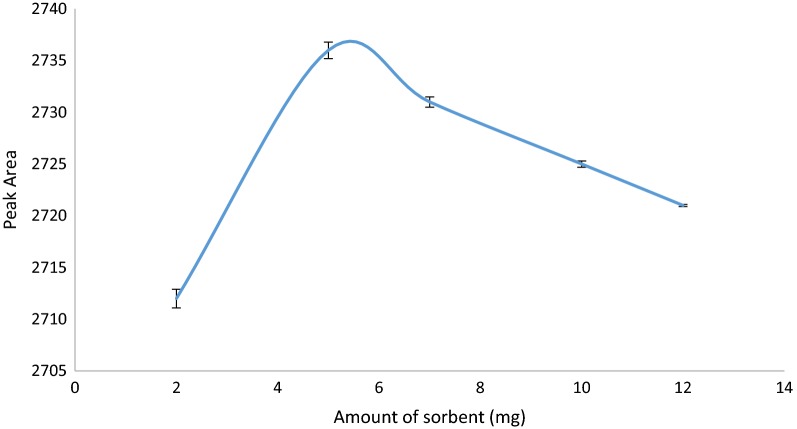
Effect of amount of sorbent on extraction efficiency of CBZ (Experimental conditions: pH: 7.5; sample volume: 150 µL; volume of eluent: 30 µL and number of draw/eject cycle for sample solution and eluent: 10 cycles)

In the pipette-tip solid phase extraction, the effect of the adsorbent amount is a main factor on extraction efficiency which must be investigated. To get the PT-SPE column more effective and at lowest possible consumption of adsorbent, different amounts of Zr-MOF-NH_2_ in the range of 2–12 mg were packed into it. As shown in Fig. 7, maximum extraction of CBZ was achieved when the amount of adsorbent increased to 5.0 mg and further increase in Zr-MOF-NH_2_ loading decrease the extraction and also prolongs the time required for sample passage. The small decrease in extraction efficiency is probably due to the fact that the quantitative desorption of CBZ from the Zr-MOF-NH_2_ became more difficult when the same amount of eluent solvent is used with the same washing cycles. Therefore, 5.0 mg was employed as packing material in the fallowing studies.

#### Effect of volume of sample solution

**Fig. 8:**
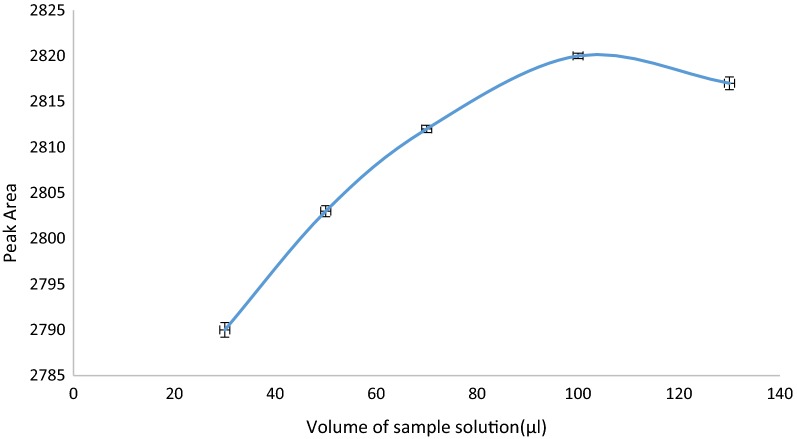
Effect of volume of sample solution on extraction efficiency of CBZ (Experimental conditions: pH: 7.5; amount of adsorbent: 5 mg; volume of eluent: 30 µL and number of draw/eject cycle for sample solution and eluent: 10 cycles)

In this regard, different volumes of sample solution (between 30 and 130 µL) were examined for the extraction of carbamazepine. As given in Fig. 8, the highest extraction efficiency was obtained when a volume of 100 µL of the sample solution was used. By increasing the volume of the sample solution, more analytes can be adsorbed on MOF sorbent; however, after a certain point, equilibrium takes place and extraction efficiency becomes constant.

#### Effect of volume of eluent

**Fig. 9:**
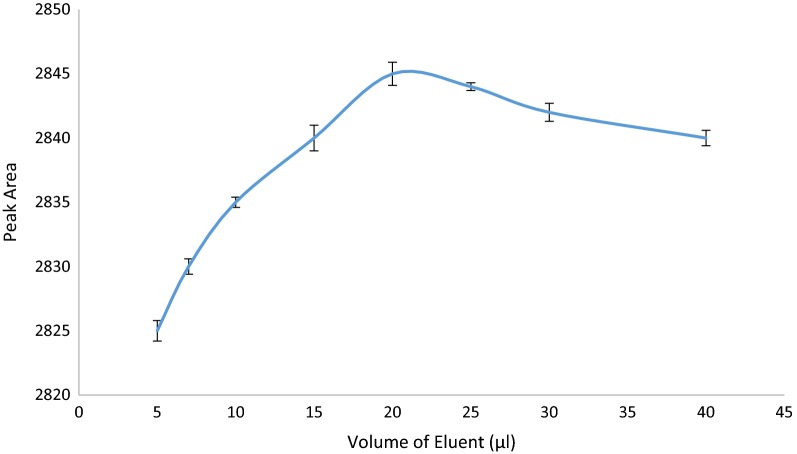
Effect of volume of eluent on extraction efficiency of CBZ (Experimental conditions: pH: 7.5; sample volume: 100 µL; number of draw/eject cycle for sample solution and eluent: 10 cycles)

In order to achieve a good enrichment factor and the highest extraction efficiency, various volume of methanol, as the eluent, between 5 and 20 µL were examined. CBZ peak area was increased with increasing the volume of eluent up to and 10 µL of methanol and then was decreased because after the optimum point, the analyte may diluted and extraction efficiency decreased (Fig. 9).

#### Effect of draw/eject of sample solution and eluent

The procedure of aspiration of a solution into pipette tip and dispensed back into the same sample tube is called aspirating/dispensing (or draw/eject) cycles, which a critical factor for PT-SPE extraction. Therefore, the influence of this parameter on the extraction efficiency was examined between 3 and 20 cycles. After 5 cycles, the extraction of CBZ from sample solution was found to be complete. Meanwhile, the best elusion of CBZ from the sorbent was occured at 7 cycles of draw/eject of eluent. In higher number of cycles, the efficiency was decreased, which is probably due to the back extraction of the analyte from adsorbent into the sample solution, causing a decrease in the recovery.

#### Reusability of the sorbent

To investigate the stability and reusability of the Zr-MOF-NH_2_ packed micro column, after desorption of CBZ from the adsorbent, the column was washed five cycles with methanol and then five cycles with deionized water. After that, several extraction and elution operation cycles were carried out under the optimized conditions. The result of experiments indicated that the adsorbent could be reused at least for eight times with a decrease of only 5% in extraction recovery. As the powder PXRD patterns of the Zr-MOF-NH_2_ before and after adsorption shown in the Fig. 3, the crystallinity of the MOF was reserved during the experimental conditions, confirming the stability of the MOF under the experimental conditions.

#### Adsorption capacity

The adsorption capacity of the Zr-MOF-NH_2_ was determined by the batch experiments. For this purpose, a standard solution containing 2000 mg L^−1^ of CBZ was applied. The amount of adsorbed CBZ was calculated by determination of difference between initial and final concentration of CBZ after adsorption. The maximum sorption capacity was defined as the total amount of adsorbed CBZ per gram of the Zr-MOF-NH_2_. The obtained capacity was found to be 32 mg g^−1^. High adsorption capacity indicated that large porosity and large surface area of adsorbent.

### Method validation

The analytical performance of the PT-SPE method was evaluated as the results shown in Table 1. Limit of detection (LOD) was obtained based on a signal-to-noise ratio of 3. The linear dynamic range (LDR) was studied by increasing concentration of the standard solution from 0.05 to 200 µg L^−1^. The repeatability of the method, expressed as relative standard deviation (RSD). Intra-day precision of proposed method was calculated for seven replicates of the standard at 50 µg L^−1^ concentration of CBZ. Repeatability was obtained 2.5% for 50 µg L^−1^ of carbamazepine. The calibration curve was obtained by plotting the peak areas of CBZ against its concentration and was linear in the range of 0.1–50 µg L^−1^ that demonstrated good linearity of proposed method. The correlation coefficient of calibration curve was 0.999.

**Table 1:** Analytical figures of merit for Zr-MOF-NH2 for extraction of CBZ

Parameter	Analytical feature
Linear Dynamic range (μg L^−1^)	0.1–50
R^2^ (determination coefficient)	0.9988
Repeatability (RSD%) (50 μg L^−1^)	2.5
Limit of detection (µg L^−1^)	0.04
Total extraction time (min)	≤ 12

### Determination of carbamazepine in real samples

**Fig. 10:**
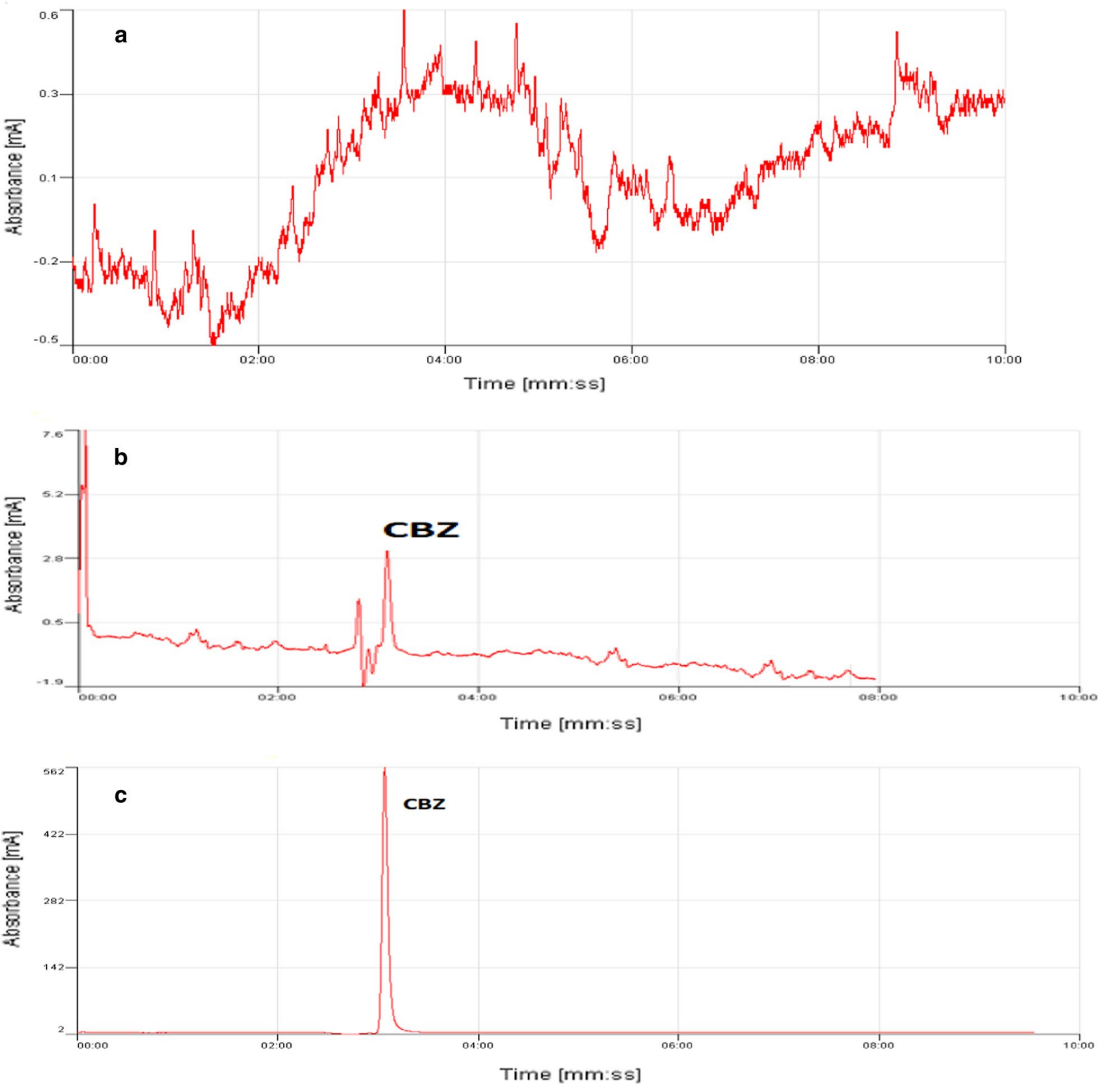
Chromatograms obtained for the analysis of carbamazepine; **a** direct injection of urine sample, **b** direct injection of urine sample spiked with 50 µg L^−1^ of CBZ, and **c** injection of spiked urine sample with 50 µg L^−1^ of CBZ after PT-SPE extraction

The proposed PT-SPE technique was successfully used for the determination of CBZ in urine and water sample. As shown in Table 2, recoveries of all spiked levels are adequate; therefore, we can use this method for the analysis of CBZ in complex matrices as urine. The chromatogram of carbamazepine in blank and spiked urine samples are presented in Fig. 10.

**Table 2:** Evaluation of carbamazepine in real samples

Sample	Concentration found (µg L^−1^)	Spiked at concentration (µg L^−1^)	Recovery (%)	RSD (%)
Urine	0	5	99.4	3.6
		20	98.8	4.2
		50	98.2	6.2
Tap water	0	50	99.2	4.7

### Comparison of proposed method with other methods

A comparison of the proposed method with those using different preconcentration techniques for CBZ determination is given in Table 3, which demonstrates the feasibility and reliability of the applied method. Shorter analysis time, lower consumption of the sorbent and sample solution, simplicity of method and lower eluent volume compared to the other SPE methods, were achieved. Also, Zr-MOF-NH_2_ as sorbent in comparison with other sorbent that mentioned in Table 3 showed high adsorption capacity, more stability and reusability.

**Table 3:** Comparisons of the proposed methods with other methods for extraction of CBZ

Methods	LOD (µg L^−1^)	LOQ (µg L^−1^)	RSD (%)	Refs.
MEPS^a^	1.5	5	267	[2]
Molecularly imprinted polymer	25	–	3.1	[13]
SBSE^b^	0.03	0.08	8.8	[45]
SPE/MGO-CD^c^	< 4	11.89	5.5	[46]
SPE/graphene- silica gel	–	0.013	–	[47]
SPE/modified sulfur nanoparticles	0.0016	0.005	3.7	[48]
SPE/oasis HLB cartridges	0.002	0.5–150	0.082	[48, 49]
Zr-MOF-NH_2_-PT-SPE	< 0.04	0.13	3.2–2.5	This study

^a^Micro extraction in packed syringe

^b^Stir-bar sorptive extraction

^c^Magnetic graphene oxide-cyclodextrin polymers

## Conclusion

A porous amino-functionalized metal organic framework containing missing-linker defects was firstly prepared and then applied for pipette-tip solid phase extraction of a drug, carbamazepine. The total time of analysis, including extraction was less than 12 min. The Zr-MOF-NH_2_ sorbent was used for at least eight extractions without any significant change in its capacity or repeatability. Only 5 mg of the sorbent was enough for filling the PT. The presence of more open active zirconium sites, more numbers of hydroxyl groups, the large porosity, very high surface area, the amino functionality, and the suitable pore size of the Zr-MOF-NH_2_ could improve the extraction of CBZ. Moreover, the fast, inexpensive, effective, reliable, applicable and organic solvent-free method can open up new practical applications for MOFs in SPE based analytical techniques.
